# Smoke-Free Recovery from Trauma Surgery: A Pilot Trial of an Online Smoking Cessation Program for Orthopaedic Trauma Patients

**DOI:** 10.3390/ijerph14080847

**Published:** 2017-07-28

**Authors:** Sam McCrabb, Amanda L. Baker, John Attia, Zsolt J. Balogh, Natalie Lott, Justine Naylor, Ian A. Harris, Christopher M. Doran, Johnson George, Luke Wolfenden, Eliza Skelton, Billie Bonevski

**Affiliations:** 1 School of Medicine and Public Health, Faculty of Health and Medicine, University of Newcastle, Callaghan, NSW 2308, Australia; amanda.baker@newcastle.edu.au (A.L.B.); John.Attia@newcastle.edu.au (J.A.); Zsolt.Balogh@hnehealth.nsw.gov.au (Z.J.B.); Luke.Wolfenden@hnehealth.nsw.gov.au (L.W.); Eliza.Skelton@newcastle.edu.au (E.S.); Billie.Bonevski@newcastle.edu.au (B.B.); 2 Hunter Medical Research Institute, University of Newcastle, New Lambton, NSW 2305, Australia; 3 Department of General Medicine, John Hunter Hospital, New Lambton Heights, NSW 2305, Australia; 4 Department of Traumatology, John Hunter Hospital, New Lambton, NSW 2305, Australia; Natalie.Lott@hnehealth.nsw.gov.au; 5 Whitlam Orthopaedic Research Centre, Ingham Institute for Applied Medical Research, Liverpool Hospital, Liverpool, NSW 2170, Australia; Justine.Naylor@sswahs.nsw.gov.au (J.N.); iaharris1@gmail.com (I.A.H.); 6 South Western Sydney Clinical School, Faculty of Medicine, University of New South Wales, Liverpool, NSW 2170, Australia; 7 School of Human, Health and Social Sciences, Central Queensland University, Brisbane, QSD 4000, Australia; c.doran@cqu.edu.au; 8 Centre for Medicine Use and Safety, Monash University, Parkville, VIC 3052, Australia; Johnson.George@monash.edu; 9 Hunter New England Population Health, Wallsend, NSW 2287, Australia

**Keywords:** smoking cessation, telemedicine, wounds and injuries, orthopedics

## Abstract

Smoking increases the risk of complications associated with orthopaedic trauma surgery, however delivery of care is low. Online interventions may provide needed smoking cessation care and promote abstinence. This study aims to examine the engagement, acceptability, and retention of an online smoking cessation program (Smoke-Free Recovery; SFR) among a sample of orthopaedic trauma patients, as well as themes around the smoking cessation process. A pilot study of SFR with 31 orthopaedic trauma patients admitted to a public hospital in New South Wales, Australia took place. Semi-structured telephone interviews were conducted following hospital discharge. Thematic analysis and descriptive statistics were used. Engagement was high with 28 participants accessing SFR during admission. Twenty individuals completed follow-up phone calls. Program acceptability was rated favourably. After discharge, changes in smoking habits were noted, with program retention low. Themes on program use included: lack of time or need for additional support; computer illiteracy or technology issues; feeling unready or too stressed to quit; or feeling they had reached the boundary of what could be learnt from the program. This study highlights the difficulties faced by patients following hospital admission, the lack of follow-up support received, and the need for consumer testing prior to roll out. Continuing to develop interventions to promote hospital-initiated cessation attempts that continue post-discharge should be a priority.

## 1. Introduction

Along with the well documented public health consequences of smoking [1,2], tobacco use is known to have negative effects on surgical outcomes including increased mortality risk [3], increased risk of post-operative infection [4], wound and flap necrosis [5], and a decrease in the tensile strength of wounds [6]. International estimates indicate that tobacco use is higher among orthopaedic trauma patients compared to the general public, ranging from 24% to 39.2% [7,8,9]. Australian figures are similar, with 37.2% of acute extremity fracture patients identified as current tobacco users in 2011 [10], twice the prevalence rates for the general population (16.1%) [11]. Further, complications after surgery often result in longer hospital stays, as well as higher rates of readmission, resulting in greater cost to the health care system [12]. The benefits of quitting either before or following surgery are clear, with a meta-analysis of randomised trials finding a statistically significant reduction in wound healing complications among former smokers when compared to current smokers [13].

A Cochrane review [14] of interventions for smoking cessation in hospitalised patients has found that intensive counselling during a hospital admission and continuing care lasting at least one month post-discharge is effective at increasing smoking cessation rates. Hospitalisation is a time which increases receptivity to stop-smoking messages [14,15,16,17], and provides an opportunity to facilitate education and behaviour change [18]. As well, the hospital admission provides patients with a smoke-free environment, presenting them with time away from their everyday triggers and a chance to experience abstinence [16,19,20]. Despite this, research indicates that patients are being given sub-optimal support for smoking cessation while in hospital [21,22,23].

There are many potential benefits to utilising an online smoking cessation program in the hospital setting. Internet delivered interventions are attractive tools for delivering messages as they are relatively low cost [24] and have the potential to reach individuals who otherwise may not have sought support, such as younger males who are less likely to seek help from professionals [25]. Behaviour change techniques such as goal setting, feedback, cognitive behaviour therapy, and motivational interviewing can be delivered using online programs, increasing their efficacy [26]. Further, online programs are beneficial from users’ perspectives with 65–80% of patients indicating that they are interested in supplementing clinician support with online programs [27,28].

There is considerable evidence for the effectiveness of online interventions for increasing tobacco smoking cessation [29,30,31]. Within the hospital setting, online programs present the additional advantage of addressing barriers often cited as reasons for not delivering smoking cessation care by staff such as: time constraints; lack of resources [32,33,34,35]; and lack of knowledge or training [34,36,37,38,39,40]. Further, internet usage among the orthopaedic trauma population has been found to be high (75–79%) [41,42] indicating the potential usefulness of an online smoking cessation program.

Given the negative health outcomes associated with continued tobacco smoking; and that the current provision of hospital smoking cessation care is low; online programs may provide a potential low-cost, low-resource intensive alternative to staff provided care. Hence, the Smoke-Free Recovery (SFR) program [43] was designed specifically for orthopaedic trauma patients to use both while in hospital and after discharge to provide patients who smoke with additional support to quit. To date, only one online smoking cessation program has been described for hospitalised patients. Harrington et al. [44] conducted a two-arm randomised controlled trial of an online smoking cessation program with three, six, and 12 months follow-ups, but found no significant differences in cessation rates. Low engagement with the online intervention was cited as a possible cause for the lack of effect, highlighting the need to develop and pilot test interventions with consumer input. To increase consumer engagement, acceptability, and retention, the SFR intervention is tailored specifically for orthopaedic trauma patients and uses interactive components, videos, and graphics to enhance engagement [45,46,47]. As well, the SFR program employs a number of behaviour change techniques (BCTs) to improve health behaviour change effectiveness, which are outlined in the development paper [43].

This paper describes the mixed methods pilot testing of the SFR online program with orthopaedic trauma patients. This study aims to examine themes relating to feasibility of the SFR program (engagement, acceptability, and retention) and themes relating to the smoking cessation process. 

## 2. Methods

*Design and setting.* A pilot study of the online SFR program was conducted with a sample of orthopaedic trauma patients. Participants were recruited from a Level One trauma public hospital in New South Wales (NSW), Australia between March and September, 2016. The hospital is a smoke-free health service where smoking is not permitted anywhere on hospital grounds. Patients who are smokers were offered nicotine patches during their hospital stay as part of hospital policy.

*Intervention.* All current tobacco smokers who agreed to trial the online program received access to SFR [43]. SFR was developed to guide users through their quit process, utilising behaviour change theory and current recommendations for smoking cessation care with hospitalised patients. The program was designed to be easy to use, requiring a grade 7 reading level (i.e., the reading level of a student in their first year of high school, aged approximately 11–12 years), and included graphics and videos. SFR contained 10 modules (Module 0: Welcome; Module 1: Recovery from surgery; Module 2: Thinking about quitting; Module 3: Quitting; Module 4: Staying quit; Module 5: Heading home; Module 6: Having trouble staying quit; Module 7: Games and quizzes; Module 8: Smoke-free diary; and Module 9: Resources) aimed to align with the individual’s hospital admission and their quit journey. Initially, the user was provided with a loading module which asked questions regarding their demographics and current level of tobacco use. This information was later used to tailor the information presented to the user by the program. After the loading module, all other modules were able to be accessed, with tailoring and branching used to allow the users to be able to revisit sections of interest as desired. Each module contained information specific to the stage of change in the quit process [48], (pre-contemplation stage aligns with immediate post-surgery recovery and the module Recovery from surgery, contemplation aligns with the module Thinking about quitting, action with Quitting, maintenance with Staying quitting, and relapse prevention with Heading home), and was designed to ask questions and provide information to increase motivation to quit.

*Participants.* Patients were eligible to pilot the program if they were: aged 18–80 years; were admitted to the hospital for a fracture (skull and cervical spine fractures not included); and were able to provide informed consent (i.e., not immediately post-operation, not suffering from post-trauma amnesia, and not suffering from a head trauma or illness which may prevent informed consent). Participants who were suffering from any transmittable disease (e.g., Methicillin-resistant *Staphylococcus aureus* [MRSA]) were also excluded. 

*Procedures.* Two methods of recruitment were used. Firstly, known eligible patients, identified by medical staff, were directly approached for inclusion in piloting SFR. Secondly, all orthopaedic trauma patients were approached as part of a separate study, which involved a health behaviour survey using a tablet device. Participants who were identified as smokers during the survey were then approached by a research assistant to determine interest. 

Initially, recruitment of patients was difficult as patients were often receiving treatment from allied health professionals during the time they were being approached by the research staff. This however was overcome by adjusting the hours worked by the recruiter, talking to the patients earlier (approximately 7 a.m. to 9 a.m.), while the staff were on their rounds rather than during practicing hours (from 9 a.m. to 5 p.m.).

**Figure 1:**
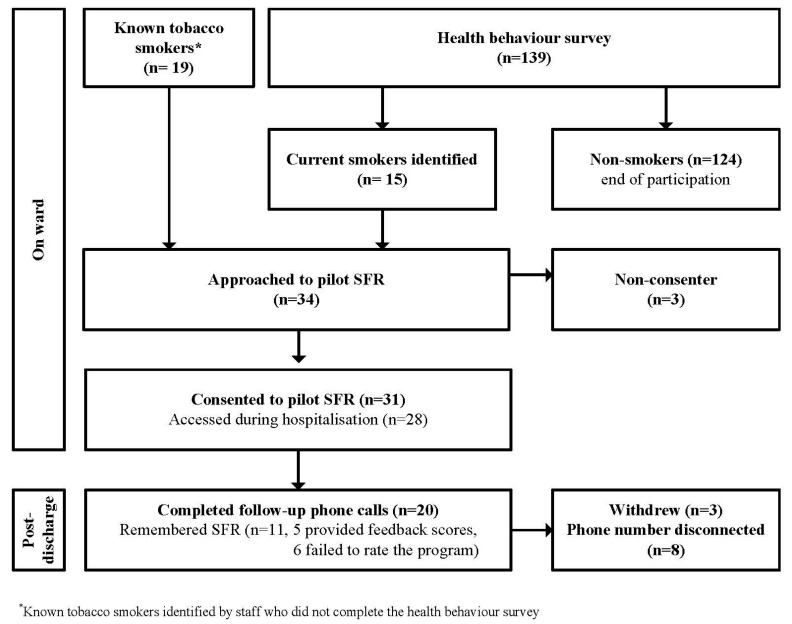
Participant recruitment flow for pilot trial of SFR.

Participants were provided with a touchscreen tablet device to use while in hospital, and a business card with the website link and their personal login information so that they were able to access the program using any internet enabled device following discharge. Figure 1 depicts the recruitment process.

All individuals were provided with an information statement and consent form specific to trialling SFR, outlining that they would have access to the program and would receive a telephone call from research staff after discharge from the hospital. Upon receipt of signed consent, participants were logged on to the SFR program using a participant identification number to de-identify their access to the program. Participants were also provided with 30 2.5 mg nicotine oral strips as part of study participation and could have received nicotine patches under hospital policy. 

Participants were contacted by telephone by a researcher at least one week and up to eight weeks after discharge. Up to 12 call attempts were made for eight weeks following discharge. All interviews were transcribed by the lead researcher. *Characteristics of participants.* For all participants, the following data were collected from their medical health records: gender; age; Indigenous status; marital status; and insurance type.

*Engagement with SFR.* Information regarding the number of modules visited and time spent on each module of the program during hospital admission were exported from the online platform. To determine engagement. Participants were also asked *“What sections of the program did you find most useful?”* (open response) to determine their engagement with the program.

*Acceptability of SFR.* Participants were first asked if they had the chance to log on to the program since being discharged (Yes; No). Those participants who had logged on to SFR were asked open-ended questions: “*What are your thoughts on the program so far?*” (open response); “*On a scale of one (not easy) to five (very easy), would you say the program is easy to use?*”. Participants who did not use the program following discharge were also asked: “*I’m interested in finding out more about the reasons around not logging on, what got in the way of you logging on?*” (open response).

*Retention with SFR.* Information regarding the number of modules visited and time spent on each module of the program after discharge were exported from the online platform. 

All participants were also asked if there was anything further they would like to comment on and if they had any questions.

*Ethics approval.* This study received approval from Hunter New England Human Research Ethic Committee (number: 14/02/19/4.04) and is registered with the Australian New Zealand Clinical Trials Registry number: ACTRN12614001147673. 

## 3. Analysis

All data were stored on secure servers at the University of Newcastle and were exported into Stata v13 (StataCorp LP, College Station, TX, USA) for analysis. Timing, and usage data from the program are presented as numbers and percentages. Qualitative analysis of the telephone interviews involved the thematic analysis of the data [49,50], with the lead review author conducting the analysis. The six phases of thematic analysis as outlined by Braun et al. [49,51] were utilised and used to determine as much information about the smoking habits and the uptake of the program as possible. NVivo v11 (QSR International Pty Ltd., Melbourne, Australia) was used to examine and determine relationships between themes. Stata v13 was used to determine all quantitative statistics.

## 4. Results 

Of 34 tobacco smokers approached to take part in this study, 31 agreed to participate (91%), with 28 individuals accessing the program during hospitalisation. Follow-up data was available for 20 (65%) individuals. Of the 11 participants who were not followed up, eight were not contactable, and three withdrew (unwilling to talk or did not want to be bothered by researcher). Demographics of those participants who provided follow-up phone calls are included in Table 1.

*Engagement with SFR.* The majority of participants (*n* = 28, 90%) used SFR during their admission. Table 2 contains a summary of the number of people who accessed each module of SFR during admission and the total number, average, and standard deviation (SD) of minutes spent within each module by all users.

Following the loading (‘Welcome’) module, where participants answered questions which tailored the information they received to their current living situations, ‘Recovery from surgery’ was the second most accessed (*n* = 22, 71%), followed by ‘Thinking about quitting’ (*n* = 11, 36%). The most amount of time was spent in the ‘Quitting’ module (average 164 min), with six people (19%) accessing it during admission.

Themes from interviews discovered that individuals engaged well with aspects of the program “*the films were very interesting especially the one of the surgeons*” [Female, 55], and that “*it* [SFR] *was enough to get me interested in quitting, I hadn’t thought of it before!*” [Male, 47].

Participants recalled specifics from the films within SFR, recalling facts they had learnt about the health effects of continuing to smoke “*Doctors tell you that smoking is bad for you but don’t go into the details. One thing I remember the most is that continuing to smoke can lead to 60% more chance of further complications.*” [Male, 26].

Some individuals did not recall access to SFR (discussed below). Of the participants who remembered using SFR in hospital (*n* = 11), SFR prompted them to make the most of their hospital admission based on the information they learned in the program. “*It was enough to get me interested in quitting, I hadn’t thought of it before... I only had a short stay but I think I would have been desperate for more help if I’d stayed longer.*” [Male, 47]. “*The program is what kept me off the smokes while I was in hospital.*” [Male, 53]. Another individual stated “*I watched one film prior to surgery… this is why I was keen to stay quit!*” [Male, 43].

*Acceptability of SFR in hospital.* Of those participants who scored SFR on ease of use (*n* = 5 participants), all said that the program was easy to use (scoring the program five out of five). Respondents stated that SFR was “*very user friendly… the information was helpful*” [Male, 44].

The remainder of the participants (*n* = 15) did not rate SFR. Of these participants, nine did not recall access during hospitalisation, while the others (*n* = 6) failed to provide a rating out of five, instead stating that the program “*had internet connection issues on the day*” [Male 47], “*it was quick loading modules*” [Male, 26], or discussing other competing issues regarding their hospital admission during their phone interview. One of the reasons for not remembering the program was medication related, with some individuals stating their medication resulted in them not clearly remembering their hospital admission (*n* = 3) “*I was on a lot of pain killers at the time.*” [Male, 60]. “*I vaguely remember using the program, I was on a lot of medication so I can’t remember specifics… I was hallucinating.*” [Female, 45]. “*I was pretty drugged up at the time. I think I saw a video, but I can’t be certain*” [Male, 23]. One individual could recall meeting the research team, but not accessing the program [Male, 29], and another accurately recalled that he was discharged prior to getting the chance to use SFR and that he had not used it since discharge [Male, 50].

One user discussed the benefits of the program’s flexibility and ability to use only those modules she was interested in: *“I looked at what I was interested in, what my main problem is. I mainly focused on the relapse prevention aspect of the program, the going home part.”* [Female, 55].

Functionality was an issue for a few during hospital admission “*The internet connection on the day was poor and that would have annoyed me had I continued to use it.*” [Male, 47]. “*It took a while to load when flipping between pages but it wasn’t enough to be annoying.*” [Male, 26]. As well, the program had some disadvantages *“Using the program was the only time I felt like smoking while in hospital”* [Male, 26].

*Acceptability of using SFR after discharge.* Reasons for which participants had not accessed the program following discharge included: were too busy; had already quit smoking; were computer illiterate or had technology problems; were not ready to quit; were too stressed; felt they had learnt enough from the program already. 

*Retention to SFR.* Few participants accessed SFR after hospital discharge (*n* = 2). These participants continued to access three modules within the program (Quitting [*n* = 2, average of eight minutes]; Games and quizzes [*n* = 2, average of two minutes]; and Smoke-free diary [*n =* 1, 1½ min]), spending on average six minutes in the program as a whole.

*The smoking cessation process.* Quitting smoking while in hospital came up often in qualitative telephone calls. All participants said that they had stopped smoking during their hospital admission. Views on quitting smoking in hospital varied, some individuals had no trouble stopping, while others reporting struggling with their forced period of abstinence. “*On admission, I was content in accepting I was going to quit, I was willing to make the most of it. I was using an e-cig to help me but it was taken off me… Not having the e-cig made me angry and frustrated, it was the only thing that helped me and made me feel like I was able to quit.*” [Female, 45].

*Stop smoking medication was also a recurring theme.* Individuals expressed interest in trying nicotine replacement therapy (NRT), the benefits they found from using NRT, common difficulties faced with using NRT (such as skin irritation and bad taste), and interest in discussing stop smoking medication with their doctor on discharge. “*I plan to visit the GP about a* [varenicline] *prescription. Talking to other people who have quit recommend this.*” [Male, 47]. “*I used those strips. They weren’t very nice, but they worked well!*” [Male, 23].

*Quitting smoking after discharge.* Participants stated that they had remained abstinent, or had cut down their smoking since discharge. Individuals reported reasons such as worrying about the health impact, financial costs, and not having the desire to smoke as why they had remained smoke-free. However, for those who had relapsed, social occasions, and cost were often cited as reasons for smoking again. “*I’ve been smoking bugger all but I did have a few* [cigarettes] *at a party the other night.*” [Male, 50]. “*I started smoking again after 2 days, it was cheaper. I went straight to the chemist to buy some more patches but they were too expensive! They were $60 for a two week pack!*” [Male, 43].

## 5. Discussion

*Engagement with SFR and smoking during admission.* This study found that there are potential benefits in providing patients with an online quit smoking program during hospitalisation, with high engagement and some users learning and utilising information to make the most of their smoke-free stay. The majority of orthopaedic trauma patients who smoked reported abstinence during their hospital admission, with changes in smoking habits evident after discharge (either remaining abstinent, or cutting down the number of cigarettes smoked per day). This indicates that the hospital admission may be effective at providing individuals with an opportunity to quit smoking and supports the idea of a teachable moment. However, these results cannot be determined as being the effect of the intervention or the hospital admission, with further research needed.

*Acceptability of SFR.* One of the modules described by users as memorable was the film of a surgeon discussing the effects that tobacco smoking can have on recovery from surgery. Such findings may reflect the salience of the information contained in the module given impeding surgery or a recency effect, given it was the first section of the program which was able to be viewed. Further, research suggests video and graphic tailored information has been found to be particularly well attended too and considered more acceptable than text [47]. As video delivered information was well received by SFR users, this may indicate that providing information using this format should be considered in future studies as a beneficial tool. Future research should continue to include brief information from hospital staff delivered in video format.

It is interesting to note that some users could not remember accessing SFR during admission, despite timing data indicating that they did log on. This may be due to medications or fogginess following anaesthesia, however, the effects of these are poorly understood [52]. Memory loss may have implications on the effectiveness of hospital based interventions. There are two ways to overcome this memory loss, provide the program prior to hospital admission (only applicable to elective patients), or provide the program after hospital admission. This too indicates that there is a need to increase post-hospital discharge care in order to overcome some of the barriers to retention of health messages learnt during hospital admission. However, given the uncertainty around the impact of medication on memory loss and memory recall, it may still be beneficial to provide individuals with information during their admission, as it may still have potential benefits to patient learning. Further research is needed to investigate the impacts of this on the feasibility of memory loss and the effects of medication on hospital based research.

*Retention to SFR and smoking after discharge.* Retention to SFR was low, with the effect of SFR on smoking cessation not determined due to the lack of control conditions. Difficulties faced by patients in using SFR following hospital admission included: lack of time; maintaining a quit attempt so not requiring additional support; computer illiteracy or technology issues; not being ready to quit; too stressed; or feeling they had reached the boundary of what could be learnt from the program. It remains important to identify ways to increase post-discharge support to address concerns which may lead to smoking relapse. The identified competing priorities in the lives of patients indicate some of the difficulties patients face when leaving hospital, with interventions needed to address and overcome these concerns. This is important as the provision of at least one month post-discharge support has been shown to be effective at increasing smoking cessation rates after discharge [14].

As found in this study, previous research has identified similar low rates of access to online smoking cessation programs after hospital discharge [44,53]. A number of factors related to the intervention may have contributed to the low rates of retention including: website design; limited tailoring especially in prompt reminders; absence of reminders; and lack of changing content [54,55,56]. Certainly, the latter was suggested from the qualitative information collected, with some users indicating that they did not continue to use the program as they had felt they had reached the boundaries of what they could learn. Additions of such techniques to promote program engagement and retention may be necessary to increase usage during and after hospitalisation. The development of future interventions should utilise different design factors to increase user engagement such as: the use of more engaging infographics [57]; increasing personalised tailoring and advice; incorporating greater interaction with clinicians [58]; the inclusion of metaphors for health behaviour change (such as a car journey); priming for desired responses (i.e., using open language to promote open-mindedness in users); and incorporating gaming design interactivity (such as the inclusion of gaming controllers) to make the program more interactive [56].

A common theme which emerged from interviews was the impact of stress and its role in relapse. For this reason, it may be important to focus future efforts on post-discharge support (especially given the relatively high number of individuals who remained quit or had cut down their smoking within the follow-up time period), focusing on aspects of patients’ lives which may result in a relapse to smoking. Additional post-discharge support may also be needed to prompt use of the online program, such as telephone counselling or email prompts. Continuing to develop interventions which provide post-discharge support is important as a Cochrane review of hospital based smoking cessation interventions indicated that those which continue at least a month post-discharge were significant at increasing the chances of making a successful quit attempt [14].

## 6. Implications for Current Practice

While the online program for continued use after discharge was underutilised, brief use of SFR during hospital admission had the desired effects, with some individuals reporting that they had learnt from the information contained in the program, possibly prompting some users to continue quit attempts after discharge. Furthermore, this study highlights that some patients were maintaining quit attempts without proactively attempting to remain smoke-free after discharge. However, it appeared that few respondents were recognising the significance of their quit attempt, or proactively attempting to maintain their quit attempt. This suggests that there is a need to promote sustained quit attempts among hospitalised patients during and after their inpatient stay. One way to do this may be to provide patients who smoke with a brief film which outlines the importance of staying quit after a hospital admission. This could include information regarding hospital smoke-free policy, and the methods of care provided to patients under it. The addition of a script for stop smoking medication on discharge may also be a potential way to increase the provision of care. Both methods would have implications for current policy as they could be incorporated into the current care provided during hospitalisation.

Additionally, the development of an online smoking cessation program for hospitalised patients should not replace what is provided as part of current hospital practice. The results of this article indicate that there are potential benefits to using an online program in the hospital setting, with engagement high, and individuals reporting to have learnt information from the program. Based on this, using an online program in conjunction with gold standard care as recommended by hospital policy may have the potential to increase the support patients receive, addressing clinical practice gaps in the provision of care. More research would be needed to determine this further, with the effectiveness of online smoking cessation program in the hospital context under-researched.

## 7. Limitations

There are some limitations to this research. All patient measures of previous and current smoking status at time of phone call were self-report, making it difficult to determine if reported rates were accurate or were influenced by social desirability bias. As well, the statistical significance of changes in smoking habits and rates on the usefulness of SFR were not ascertained in this study. Furthermore, there was no control group included in this study. Despite this, the assessments were conducted by research staff independent of the treating team and patients were aware their responses would not affect their treatment. During interviews participants were willing to discuss their hospital admission and smoking habits, reflecting this. Further, while biologically confirmed smoking status is the gold standard measure, it was deemed too intrusive given the recruitment difficulties this study initially had.

## 8. Conclusions

Smoking rates are high in the orthopaedic trauma population. Online programs are acceptable yet few people were found to engage with the program beyond several minutes duration and the most pertinent content. When discharged, no further usage was seen, however, the addition of prompts may assist maintaining retention. Therefore, a possibly useful next step would be to offer modules that are most relevant during hospital admission along with proactive referral to quitline for the duration of hospital admission. Intensive post-discharge support needs to be provided, and should address the competing priorities in the lives of orthopaedic trauma patients.

## Figures and Tables

**Table 1:** Demographics of individuals who completed follow-up phone calls.

Demographic Characteristics	*n* = 20 (%)
Gender	
Male	14 (70%)
Female	6 (30%)
Age mean (SD)	47.9 (14.3)
Fractured type	
ankle/leg	6 (30%)
Hand/wrist/elbow/arm	4 (20%)
NOF */femur/pelvis	6 (30%)
Multiple fractures	4 (20%)
Insurance type	
No insurance	15 (75%)
Private insurance	5 (25%)
Indigenous status	
Non-indigenous	19 (95%)
Indigenous	1 (5%)
Country of Birth	
Australia	17 (85%)
Other	3 (15%)
Marital status	
Single	10 (50%)
Married/defacto/Partner	7 (35%)
Widowed/divorced/Seperated	3 (15%)

* Neck of Femur fracture.

**Table 2:** Number of people, total and average time in minutes spent in each module during admission (*n* = 31).

Module	Number (%)	Total Minutes	Time per Participant Mean (SD)
Welcome	28 (90%)	- *	- *
Recovery from surgery	22 (71%)	81	4 (5.6)
Thinking about quitting	11 (36%)	102	9.5 (8.1)
Quitting	6 (19%)	164	27.4 (24.7)
Staying quit	6 (19%)	21	3.4 (2.0)
Heading home	5 (16%)	49	9.8 (11.2)
Having trouble staying quit	3 (10%)	48	15.9 (9.5)
Games and quizzes	6 (19%)	44	7.5 (6.7)
Smoke-Free Diary	3 (10%)	80	26.9 (40.6)

* As this was the loading module, timing information was not calculated.
